# Intestinal Permeability of Artesunate-Loaded Solid Lipid Nanoparticles Using the Everted Gut Method

**DOI:** 10.1155/2018/3021738

**Published:** 2018-04-30

**Authors:** Wadzanayi L. Masiiwa, Louis L. Gadaga

**Affiliations:** Drug and Toxicology Information Service (DaTIS), School of Pharmacy, College of health Sciences, University of Zimbabwe, P.O. Box A178, Avondale, Harare, Zimbabwe

## Abstract

**Background:**

Artesunate is one of the most potent, rapidly acting and therapeutically versatile antimalarial drugs. Its efficacy is hampered by poor aqueous solubility and stability resulting in low oral bioavailability. Recent efforts to nanoformulate artesunate have shown great potential of improving its dissolution profile and bioavailability. However, no study has yet been done to investigate the intestinal permeability of these nanoformulations, which is a critical determinant of systemic absorption.

**Objective of the Study:**

The main aim of the study was to determine the intestinal permeability of artesunate-loaded solid lipid nanoparticles (SLN).

**Method:**

The microemulsion dilution technique was used to fabricate artesunate-loaded solid lipid nanoparticles.* In vitro* drug release studies were performed at pH 1.2 and 6.8 using the dialysis membrane method. The everted gut sac method was used to assess the intestinal permeability of the prepared nanoparticles.

**Results:**

The average particle size was 1109 nm and the polydispersity index (PDI) was 0.082. The zeta potential was found to be −20.7 mV. The encapsulation efficiency of the solid lipid nanoparticles obtained was 51.7%. At both pH 1.2 and 6.8, pure artesunate was rapidly released within the first 30 mins while the SLN showed a biphasic release pattern with an initial burst release during the first hour followed by a prolonged release over time. The rate of drug release increased with increasing pH. The apparent permeability (*P*_app_) of SLN was found to be greater (0.169 mg/cm^2^) as compared to that of pure artesunate (0.117 mg/cm^2^) at the end of the experiment.

**Conclusion:**

The results obtained in this study showed that the microemulsion dilution technique can be used to formulate artesunate solid lipid nanoparticles. The formulation exhibited a sustained drug release profile. The intestinal permeability of artesunate could be enhanced by the nanoformulation.

## 1. Introduction

Parasitic diseases pose a major global public health problem, particularly in developing countries. Amongst these diseases, malaria continues to be the most prevalent and debilitating in humans [1]. It is endemic in 107 countries worldwide, with a global incidence of 402 million [2]. Tropical countries are more prone to the disease which claims approximately 1-2 million deaths annually [3]. The burden is higher in sub-Saharan Africa accounting for 88% of all malaria cases and 90% of all malaria-related deaths. In Zimbabwe, malaria continues to be a major health concern with almost 50% of the population (7.1 million) at risk of contracting malaria [4].

Antimalarial drugs have been indispensable in the fight against malaria. However, the emergence of plasmodium resistance has rendered drugs like chloroquine and sulfadoxine-pyrimethamine (SP) that were used over the years ineffective [5]. Today, the World Health Organisation (WHO) recommends the use of artemisinin derivatives in combination with other antimalarials as first-line treatment for malaria [3]. Amongst these derivatives, artesunate is the most therapeutically versatile drug used in mild to severe malaria [6]. It has the greatest antimalarial activity compared to other artemisinins [7] and has also been extensively documented for its strong cytotoxicity against cancerous cells [8]. Despite its wide therapeutic use, artesunate exhibits variable pharmacokinetics which subsequently compromises its optimal therapeutic efficacy.

**Figure 1:**
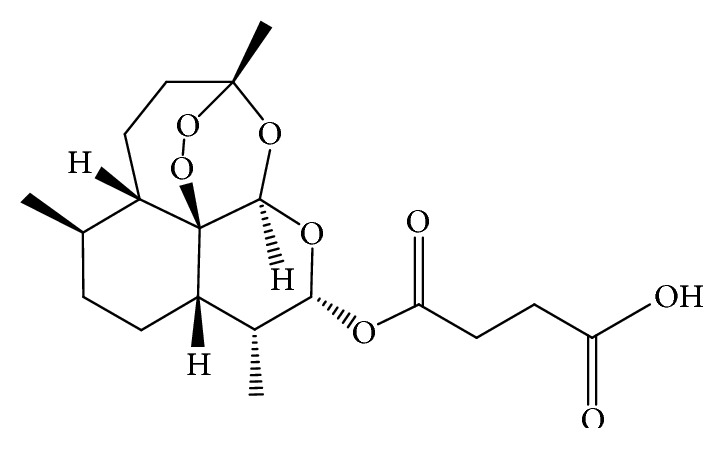
Chemical structure of artesunate.

Artesunate is poorly soluble in acidic and neutral conditions and has an aqueous solubility of 56.2 *μ*g/ml [9]. It also has a relatively low oral bioavailability of approximately 40% [10, 11]. It is highly unstable in aqueous acidic and neutral conditions [12, 13]. Its active metabolite (DHA) is rapidly decomposed to the inert end product, deoxyartemisinin, and is also easily reduced by biological molecules at physiological pH 7.4 [14]. Artesunate (C_19_H_28_O_8_) is also a weak acid (Figure 1); hence its solubility, dissolution, and permeability are affected by altered pH conditions. At acidic pH, artesunate is very poorly soluble [9, 15].* In vitro* studies have also revealed that the permeability of artesunate at pH 7.4 is 8-fold lower than that of artemisinin. As a result, in an* in vivo* situation, site-dependent absorption may occur and subtherapeutic concentrations may eventually reach the systemic circulation [16].

Continued parasite exposure to suboptimal antimalarial activity risks the development of resistance. Recent alarming reports from Japan, Thailand, Zanzibar, and Greater Mekong region have revealed artemisinin treatment failure [2] (Saunders et al., 2012), which is also the same area from which chloroquine and SP resistance initially developed [17]. Henceforth, there is a high possibility that this resistance problem could spread to all parts of the world, derailing efforts that have been made to eliminate malaria and triggering increased cases, deaths, and healthcare costs to both the victims and the government.

The threat of the spread of artemisinin resistance is prompting development of strategies to optimise the current antimalarial treatment [4]. Although other strategies to optimise artemisinin drug delivery have been investigated [18], one of the potentially promising strategies is the use of nanomedicine drug delivery systems (NMDDS) which exhibit exceptional characteristics such as target specificity, increased solubility, protection from acid degradation, and a large surface to volume ratio which allows drugs to penetrate through cell and tissue barriers [19, 20].

Researchers have widely explored the potential of some NMDDS such as liposomes in improving therapeutic effectiveness of artesunate. Results have shown improved solubility, bioavailability, and a sustained drug release which could prolong the drug's blood circulation and boost parasite exposure to the drug leading to enhanced absorption and antimalarial activity [21]. However, the liposomes were only stable at pH 5 for a short period of ten days [21]. Solid lipid nanoparticles offer increased stability and minimise drug leakage [22, 23]; hence incorporation of artesunate in SLN could be a more effective approach.

However, the overall absorption of orally administered drugs depends on a critical rate-limiting step, which is its permeability through the intestinal epithelium [24]. A wide research has thus been done on the permeability of nanoparticles over the past years which correlated with improved therapeutic activity [25, 26]. Despite all these endeavours, no study has yet been done to investigate the intestinal permeability of artesunate nanoparticles. Henceforth, it is imperative to study the impact of this factor on the subsequent rate and extent of absorption of artesunate SLN. Thus the objective of the current study was to formulate artesunate solid lipid nanoparticles and to determine their ex vivo permeability using the everted gut sac method.

## 2. Materials and Methods

### 2.1. Chemicals and Equipment

Glyceryl monostearate was purchased from Sigma Aldrich (Germany). Artesunate was a donation from Parirenyatwa Group of Hospitals (Harare, Zimbabwe). Tween 80 and n-butanol were obtained from Sky Laboratories (Harare, Zimbabwe). Methanol, buffer salts, and all the other reagents were obtained from School of Pharmacy laboratories (University of Zimbabwe). Equipment which was used include the following: UV-Visible Spectrophotometer (UV-1601 Shimadzu, Japan), Dissolution Unit (VK 7000 Vankel Technology Group, North Carolina), Ultrasonic Bath (model 702, South Africa), Magnetic Hot Plate Stirrer (Lasany), and a Homogenizer (Ultra-Turrax T10 basic).

### 2.2. Preparation of Solid Lipid Nanoparticles

Artesunate solid lipid nanoparticles were prepared using the modified microemulsion dilution technique [27, 28]. Glyceryl monostearate (500 mg) was melted in a beaker at 70°C on a water bath. Artesunate (100 mg) was added to the lipid melt under continuous stirring using a stirring rod. An aqueous emulsifier mix containing 3.4 ml Tween 80 (surfactant), 1.1 ml n-butanol (cosurfactant), and 2.1 ml distilled water was stirred at a speed of 300 rpm and at a temperature of 70°C on a hot plate magnetic stirrer. The emulsifier mix was then added to the drug-lipid melt under mild mechanical stirring. The hot microemulsion formed was added dropwise using a beaker to ice-cold water (0–5°C) in the ratio (1 : 5) and homogenized for 1 hour at 9000 rpm. The solid lipid nanoparticles dispersion formed was subjected to ultrasonication for 10 minutes and then freeze dried for 24 hours. Blank solid lipid nanoparticles were prepared using the same technique without adding the drug (artesunate).

### 2.3. Construction of a Calibration Curve

A standard stock solution (0.1 mg/ml) was prepared by dissolving 10 mg of artesunate in 100 ml of methanol. A series of known concentrations (0.03–0.09 mg/ml) were prepared from diluting the standard stock solution. The absorbance of the serial dilutions was measured in triplicate using an UV-Visible Spectrophotometer at 240 nm and used to construct a calibration curve (concentration versus absorbance). The method was validated by determining the accuracy, precision repeatability. The equation of the calibration curve, *y* = *mx* + *c*, was used to determine the unknown concentrations of artesunate.

### 2.4. Encapsulation Efficiency (EE) and Drug Loading (DL)

The yield obtained was determined by weighing the SLN on an analytical balance. To determine the encapsulation efficiency, 100 mg of artesunate solid lipid nanoparticles was dissolved in 10 ml methanol and sonicated for 10 min to completely rupture the lipid. The resultant solution was filtered using a 0.45 *μ*m membrane filter. The concentration of the drug in the filtrate was determined by measuring its absorbance against methanol using a UV spectrophotometer at 240 nm. The following equations were then used to calculate the encapsulation efficiency and drug loading:

(1) EE%=Practical  drug  loadingTheoretical  drug  loading×100%,DL%=Amount  of  drug  present  in  nanoparticlesTotal  amount  of  lipid  nanoparticles×100%.

### 2.5. *In Vitro* Drug Release

In vitro release properties were studied using the dialysis tube method. Artesunate SLN equivalent to 2 mg of pure artesunate was dissolved in 5 ml HCl buffer pH 1.2 which was prepared by dissolving 7.455 g of KCl in 500 ml distilled water and then adding 850 ml of 0.2 M HCl. A dialysis tube was filled with the resultant solution and the ends were secured with a thread. The same procedure was done with 2 mg of pure artesunate. The dialysis tubes were suspended on a paddle of the dissolution apparatus with a thread and immersed in 500 ml of phosphate buffer at pH 1.2. The paddle was continually stirred at a speed of 100 rpm and the temperature was maintained at 37°C. Samples were withdrawn at predetermined time intervals and replaced with an equivalent volume so as to maintain sink conditions. The concentration of the drug was determined by measuring its absorbance using a UV spectrophotometer at 240 nm. The procedure was repeated using a phosphate buffer at pH 6.8. The following equation was then used to calculate the cumulative percentage release:

(2) Amount  of  drug  released mg/ml=Conc. ×Dissolution  bath  volume×Dilution  factor1000,Percentage  drug  release%=Amount  of  drug  released mg/mlInitial  amount  of  drug  in  dialysis  tube mg/ml×100%,Cumulative  percentage  release=Volume  of  sample  withdrawn mlBath  Volume ml×%  tn−1+%  tn,

where (%)  *t*_*n*−1_ is percentage release previous to “*t*” and (%)  *t*_*n*_ is percentage release at time “*t*”.

### 2.6. Apparent Permeability

The everted gut sac method was used to determine the apparent permeability of artesunate SLN [26, 29]. Male rats weighing 200–250 g were fasted for 24 hours with access to water. The rats were anesthetized and a midline abdominal incision was made to isolate the intestines. The jejunum was cut into equal segments, flushed with normal saline to remove the contents, and then immersed in ice-cold Krebs solution that was pregassed with carbogen. Each segment was inverted by gently pushing a notched glass rod through the whole length of the intestine and then filled with 1 ml of Krebs solution. Both ends of each segment were secured with a thread forming an everted gut sac. Each sac was immersed in Krebs solution containing artesunate SLN equivalent to 5 mg of pure artesunate at 37°C. Samples were withdrawn at predetermined time intervals from the inside of the sac. The concentration of the drug was determined by measuring its absorbance using a UV spectrophotometer at 240 nm. The procedure was repeated using 5 mg of pure artesunate. The following equation was then used to calculate the apparent permeability:

(3) Apparent  permeability mg/cm2=Conc.×VolumeMucosal  surface  area.

To calculate the mucosal surface area, the intestine was considered a cylinder and the following equation was used:

(4) Mucosal  surface  area cm2=circle  circumference  π  diameter×intestine  length cm.

To calculate the apparent permeability coefficient, the following equation was used:

(5) Apparent  permeability  coefficient cm/s=dQdt×A×C°,

where *dQ*/*dt* is the rate of drug diffusion from the basolateral side, *A* is the mucosal surface area, and *C*° is the initial concentration of the drug on the apical side.

The mean mucosal surface area *A* was 7.85 cm^2^ and the initial concentration of the drug in the donor compartment (*C*°) was 0.5 mg/ml and *dt* was 900 seconds.

Concentration of pure drug in the intestine at 900 seconds (*dQ*) = 0.113 mg/ml.

Concentration of artesunate nanoparticles at 900 seconds (*dQ*) = 0.199 mg/ml.

Hence the coefficient calculations for the pure drug was 3.20 × 10^−5^ cm/s and 5.63×10^−5^ cm/s for artesunate nanoparticles.

### 2.7. Statistical Analysis

Data obtained from the drug release and permeability studies was evaluated using the GraphPad Prism 7.0 software and a paired *t*-test was performed to determine the statistical differences in the profiles. The level of significance was set at *p* < 0.05. The model independent approach was used to assess the similarities in the dissolution profiles of the graphs.

## 3. Results

### 3.1. Calibration Curve of Artesunate at 240 nm

The standard curve of concentration versus absorbance of artesunate showed a linear relationship which satisfied the equation *y* = 7.870*x* − 0.1829. The equation was used to determine the unknown concentrations of artesunate.

### 3.2. Encapsulation Efficiency and Drug Loading

The preparation method yielded 2120 mg of the nanoparticles. The encapsulation efficiency of artesunate solid lipid nanoparticles prepared using the microemulsion dilution technique was 51.7% and the drug loading was 2.44%.

### 3.3. Particle Size and Zeta Potential

**Figure 2:**
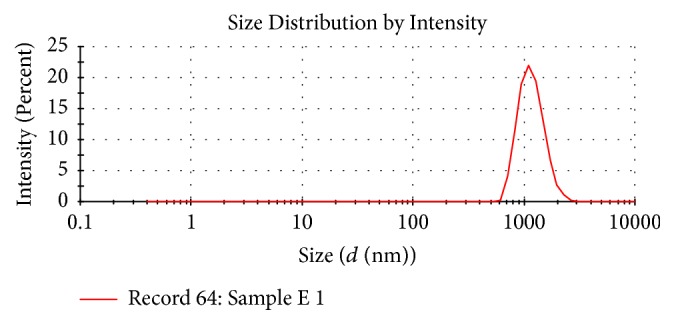
Particle size distribution of* artesunate* SLN.

The average particle of artesunate SLN was 1109 nm with a PDI of 0.082 as shown in Figure 2. The zeta potential of the artesunate SLN was found to be −20.7 mV as shown in Table 1.

### 3.4. *In Vitro* Drug Release at pH 6.8 and pH 1.2

**Figure 3:**
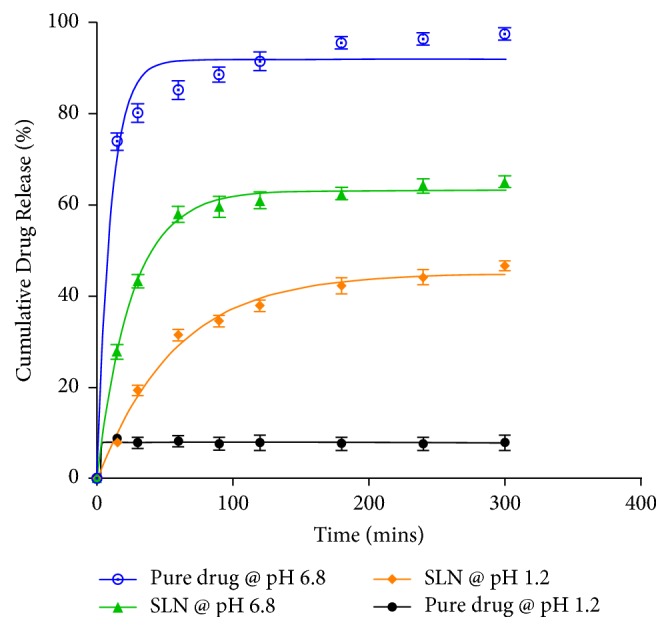
*In vitro* drug release study of pure artesunate and artesunate SLN at pH 6.8 and pH 1.2.

At pH 6.8, 75% of the pure drug (artesunate) was rapidly released in the first 15 minutes and it gradually increased to a maximum of 96.85% after 3 hours as shown in Figure 3. Compared to the pure drug, the release of artesunate SLN (29.25%) was lower in the first 15 minutes and it gradually increased to a maximum of 63.64% after 4 hours, which is almost 2/3 of the maximum release of the pure drug. The gradient of the graph of pure artesunate (~0.04709) was also steeper than that of the solid lipid nanoparticles (~0.01934). The SLN demonstrated an initial burst release of artesunate SLN of 58.57% in the first hour followed by a steady release in the subsequent 3 hours. Comparing the two graphs (pure drug versus SLN), there was a significant difference in the release profiles with a *p* value of 0.0004.

In acidic pH (pH 1.2), there was a rapid release of 8.72% of the pure drug in the first 15 minutes and it reached a maximum of 8.8% within 30 minutes as shown in Figure 2. The release remained steady over the subsequent 270 minutes. Compared to the pure drug, the release of SLN was greater and it reached a maximum of 46.08% in 5 hours (300 minutes), which is almost five times that of the pure drug. The SLN exhibited an initial burst release of about 30% in the first hour followed by a steady release in the next 4 hours. The gradient of the graph of the pure drug (~0.5420) was also steeper than that of the SLN (~0.007445). Comparing the two graphs (pure drug versus SLN), there was a significant difference in the release profiles with a *p* value of 0.0022.

### 3.5. Intestinal Permeability

**Figure 4:**
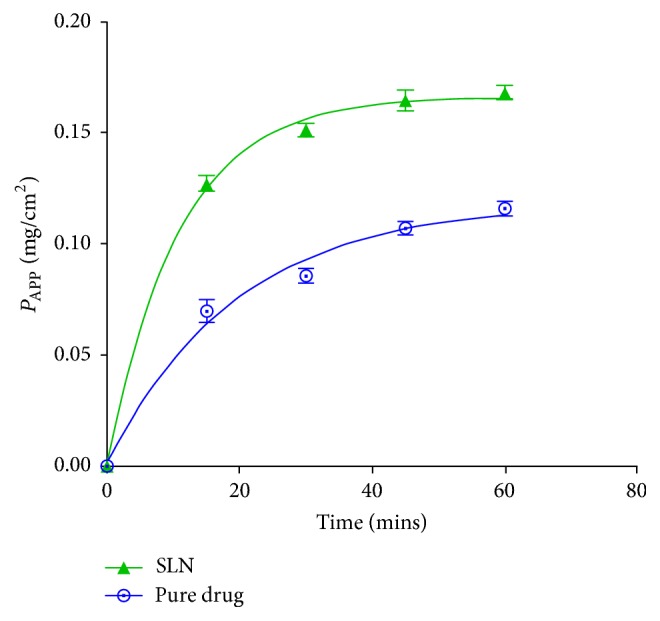
Comparison of the apparent permeability of pure artesunate and artesunate SLN at pH 7.4.

From the graph shown in (Figure 4), the apparent permeability for both the pure drug and the SLN followed a similar pattern over time. The apparent permeability of the pure drug gradually increased from 0.072 mg/cm^2^ at 15 minutes to a maximum of 0.118 mg/cm^2^ over the subsequent 45 mins. Compared to the pure drug, the apparent permeability of SLN was greater and it gradually increased from 0.127 mg/cm^2^ at 15 mins to a maximum of 0.166 mg/cm^2^ over the subsequent 45 mins. The gradient of the graph of SLN (~0.0409) was almost double that of the pure drug (~0.02341). Generally, higher permeation was observed at each time point and the apparent permeability coefficient of the SLN (5.63 × 10^−5^ cm/s) was significantly higher than that of the pure drug (3.20 × 10^−5^ cm/s) with a *p* value of 0.0171.

## 4. Discussion

The development of NMDDS in particular SLN has demonstrated great potential of improving the therapeutic efficacy of drugs with poor aqueous solubility and permeability. Artemisinins are the mainstay of malaria therapy whose optimal effectiveness is hindered by poor solubility, instability, and pH-dependent permeability [10, 16]. The use of SLN could be an effective strategy of improving the intestinal permeability of artemisinins, which boosts parasite exposure to these drugs and reduces the risk of the emergence of resistance. Henceforth, the principal goal of this study was to investigate the intestinal permeability of artesunate SLN.

Artesunate solid lipid nanoparticles were prepared using a modified microemulsion dilution technique. A preformulation study is normally done to screen for optimum formulation parameters mainly the nature and amount of the lipid, surfactant, and cosurfactant based on particle size, zeta potential, and encapsulation efficiency. However, due to limited resources, screening was only done for the lipid in this study based on physical appearance and sedimentation rate. The relative concentrations of lipid, surfactant, and cosurfactant were adapted from a study by Boonme and colleagues [27].

Glyceryl monostearate (GMS) and tristearin were the two lipids that were screened for suitability to the microemulsion method using the same concentrations. Microemulsions are clear, transparent thermodynamically stable dispersions [30]. On visual analysis, tristearin formed a milky dispersion whereas GMS formed a clear transparent dispersion. Moreover, the nanosuspension of tristearin-based SLN formed a sediment within 24 hours of storage at room temperature, whereas GMS-based SLN remained stable. This could be explained by the different structures of the lipids. Lipids with short hydrocarbon chains and polar functional groups such as GMS have smaller molecular weight volumes that can easily penetrate the hydrocarbon portion of the surfactant interface forming a microemulsion compared to triglycerides such as tristearin [27]. Triglycerides also form a perfect lattice which causes expulsion of solubilised drugs over time compared to mono- and disaccharides [31, 32]. Therefore, GMS was selected as the lipid to be used in this study.

Encapsulation efficiency (EE) refers to the amount of drug successfully entrapped within nanoparticles relative to the amount that was initially loaded. This parameter is highly dependent on the concentration of the lipid, surfactants, and the drug-lipid ratio [33].

The encapsulation efficiency of artesunate SLN obtained in this study was 51.7%. This is slightly higher compared to recent studies done using polymer-based artesunate nanoparticles by Dauda et al. [8], who obtained an EE of 38.4 ± 10.1%. This could be related to the preparation conditions. Polymeric nanoparticles are prepared using high concentrations of organic solvents. However, artesunate is highly unstable in aqueous conditions [12] and hence may be degraded by these solvents, leading to drug losses during preparation. Solid lipid nanoparticles avoid the use of organic solvents improving the physical stability of artesunate leading to fewer drug losses [23].

On the contrary, similar studies on the production of SLN using the microemulsion method usually produce higher EEs above 80% [28, 32, 34]. The discrepancy could have been caused by the higher drug-lipid ratio (1 : 5) and lower lipid content (7%) used in this study as compared to other previous studies. An increase in the lipid concentration creates more accommodation space of the drug and improves solubilisation of the drug in the lipid. The viscosity of the medium is also enhanced which causes a faster solidification of the SLN, limiting the migration of the drug into the aqueous phase [33]. However, raising the lipid content above 5–10% has a risk of increasing the particle size [35].

The ability of the lipid to dissolve or disperse the drug also has an effect on the EE. Hydrophilic drugs generally have a low affinity for the lipid; hence they have a tendency of migrating to the aqueous phase during preparation compromising the EE [36]. Artesunate is a water-soluble drug which might have resulted in the relatively low EE obtained.

The two most important characteristics of SLN are particle size and distribution which affect the rate of drug release, biodistribution, mucoadhesion, and diffusion across the epithelial membranes of the gastrointestinal system [37]. The average particle size of artesunate SLN obtained in this study was 1109 ± 36.43 nm. This was larger compared to a similar study done by Boonme and colleagues who obtained a mean particle size of 566.7 ± 540.2 nm. This can be highly attributed to the changes in stability that occurred during storage of two months and transportation to South Africa for analysis. This exposed the formulation to high temperatures. The stability of SLN is highly dependent on the surfactant which prevents aggregation of particles and requires refrigerated conditions to remain stable. High temperatures cause a reduction in the microviscosity of the surfactant resulting in the destabilization of the system which promotes particle aggregation. The kinetic energy of SLN also increases at high temperatures overcoming the electrostatic repulsive forces between particles leading to particle agglomeration [38]. The increase in particle size could also have been caused by freeze drying. The process generates mechanical stress on the SLN leading to the destabilization of the colloidal system which facilitates particle growth [39].

The size distribution can be expressed either as a graphic or as polydispersity index (PDI). The values of PDI generally range from 0 to 1. Monodisperse samples have values close to 0 whereas polydisperse ones have them closer to 1 [40]. Polydispersity index values less than 0.3 are accepted as the optimum value by most researchers [41]. The PDI obtained in this study was 0.082 which indicated that the SLN were homogenously distributed.

The zeta potential (ZP) is an important parameter which measures the surface charge of nanoparticles [42]. It gives information on the magnitude of electrostatic attraction or repulsion between particles in an aqueous colloidal dispersion [43] which allows prediction of the storage stability of aqueous dispersions [35, 44]. High ZP values (more than 25 mV or less than −25 mV) are required to stabilize colloidal dispersions. Generally, SLN have a negatively charged surface due to the negative charge of lipids and nonionic surfactants that are often used [28]. The zeta potential obtained in this study was −20.7 mV which is slightly lower than the expected values (more than 25 mV or less than −25 mV). The surface coverage of the nanoparticles by a nonionic surfactant such as Tween 80 reduces their electrophoretic mobility which could have resulted in a lower zeta potential. However, a low zeta potential is not conclusive evidence that the nanoparticles were unstable because nanoparticles can be sterically stabilized by surfactants despite having a low zeta potential [32, 38], (Shah et al., 2014).

The sole purpose of utilizing NMDDS is to enhance drug delivery (Pal et al., 2011). One way of assessing the manner and extent of drug delivery is to conduct* in vitro* drug release studies. The nature of the lipid matrix, concentration of surfactant, solubility of drug in the lipid phase, and the production parameters all have an influence on the release profile of SLN [45]. The model of drug incorporation in the SLN also significantly affects the mechanism of drug release from the SLN.

In this study, the drug release properties of artesunate SLN versus the pure drug were investigated in simulated gastric fluid (pH 1.2) and intestinal fluid (pH 6.8). A rapid release of pure artesunate within the first 30 minutes was exhibited at both pH conditions whereas the SLN followed a biphasic release pattern with an initial burst release in the first hour followed by a prolonged release of the drug over time (Figure 2). The biphasic release can be explained by the drug distribution in the SLN. High temperatures employed in the microemulsion method increase the solubility of the drug in the water phase causing it to partition from the molten lipid to the aqueous phase. Upon rapid cooling, a fraction of the drug is incorporated into the lipid core while the remaining accumulates in the shell comprising a micellar coat [23, 46]. The drug adsorbed on the surface micellar coat account has a shorter diffusional distance which could have caused the initial burst release observed [28]. Additionally, the initial release can also be explained by the larger specific area of the nanoparticle and the accelerating effect caused by the high surfactant concentrations [23, 32]. The subsequent prolonged release can be attributed to the drug localised within the lipid core. The core of SLN remains solid at body temperature which lowers the mobility of the drug, hence slowing down the release of the encapsulated drug [23, 47].

Generally, a faster drug release was observed with increasing pH (from pH 1.2–pH 6.8) in both the pure drug and SLN. This can be largely attributed to the physicochemical properties of the drug under study (artesunate). Artesunate is a weak acid; hence it exhibits pH-dependent solubility and stability. At acidic pH, artesunate is very poorly soluble [9, 15]. Its solubility increases with pH which could explain the increase in the rate of drug release from pH 1.2 to pH 6.8 observed in this study [9]. Moreover, artesunate contains a free carboxylic acid group which makes it susceptible to acid hydrolysis [48]; hence it is highly unstable under aqueous acidic conditions [12, 13]. However, the rate of drug release from the SLN was fivefold greater than that of the pure drug at pH 1.2. This might be attributed to the fact that SLN have the ability to protect acid-labile and sensitive compounds such as artesunate from the outer gut environment and to improve the solubility of the incorporated drugs [31, 44].

Intestinal permeability refers to the ability of a compound to penetrate through the intestinal epithelium. It is an important factor of the absorption of SLN [26]. The capacity of SLN to pass through the intestinal barrier is mainly governed by their size, solubility, composition, and charge. Results obtained in this study revealed that the formulated SLN had greater permeability than pure artesunate. This is consistent with results obtained in previous studies where sulpiride and *γ*-tocotrienol SLN enhanced the intestinal permeability of the respective drugs [25, 26]. Improvement in the intestinal permeability has been largely attributed to the tiny size of SLN which increases contact surface area and prolongs drug residence time. This facilitates adhesion to the gut mucosal layer and penetration into the intervillous space leading to a higher diffusion rate of the drug [24, 26, 49]. In addition, reduction in particle size can cause augmented dissolution and saturation solubility which elevates the concentration gradient between the intestinal epithelial cells and the underlying mesenteric circulation resulting in improved drug absorption [26]. Emulsifiers used in the formulation could also have contributed to the enhanced permeability because they are surface-active agents capable of altering membrane fluidity leading to improved drug absorption across the gut [49].

## 5. Conclusion

According to the results obtained in this study, the microemulsion dilution technique can be used to fabricate artesunate-loaded solid lipid nanoparticles. The formulation exhibited a sustained drug release profile which could prolong the drug residence time in the blood and boost parasite exposure to the drug, hence limiting the development of resistance. The permeability of artesunate was also significantly enhanced and this could improve the rate and extent of absorption of artesunate resulting in improved therapeutic efficacy.

## 6. Future Work

The everted gut sac method is simple, cheap, and easy to conduct but there is a risk of morphological damage during preparation of the gut sac. Hence there is need to conduct a similar study using other methods such as the in situ intestinal perfusion or loop method and compare the results. There is also a need to conduct an* in vivo *study which integrates the functional systems of the whole body such as enzymatic system (lipases), hepatic metabolism, and lymphatic transport that could affect the fate of artesunate SLN.

Cryoprotectants such as glucose can also be added during freeze drying to prevent particle aggregation during the process and upon storage. Other forms of lipid-based delivery systems such as nanostructured lipid carriers (NLC) can also be used which minimise drug expulsion from the lipid matrix by forming an amorphous stable structure which could improve the encapsulation efficiency.

Preliminary studies that include varying lipid and surfactant ratios can be carried out to optimise the encapsulation efficiency and particle size obtained. Other characterisation techniques such as microscopy can also be carried out to understand the morphology and shape of SLN.

## Figures and Tables

**Table 1:** Particle size distribution and zeta potential of Blank and artesunate SLN.

	Average Particle size	PDI	Zeta Potential
Blank SLN	1478 ± 60.28	0.306 ± 0.17	−11.1 ± 1.10
Artesunate SLN	1109 ± 36.43	0.082 ± 0.06	−20.7 ± 0.92

## Abbreviations

SLN: Solid lipid nanoparticles
PDI: Polydispersity index
*P*_app_: Apparent permeability.
